# Four‐dimensional oral health‐related quality of life impact in children: A systematic review

**DOI:** 10.1111/joor.13066

**Published:** 2020-08-19

**Authors:** Maisa Omara, Tanja Stamm, Katrin Bekes

**Affiliations:** ^1^ Section for Outcomes Research Center for Medical Statistics, Informatics, and Intelligent Systems Medical University of Vienna Austria; ^2^ Ludwig Boltzmann Institute for Arthritis and Rehabilitation Vienna Austria; ^3^ Department of Pediatric Dentistry University Clinic of Dentistry Medical University of Vienna Austria

**Keywords:** adolescent, child, oral health, patient‐reported outcome measures, quality of life, systematic review

## Abstract

Oral health‐related quality of life (OHRQoL) is an important dental patient‐reported outcome which is commonly based on 4 dimensions, namely Oral Function, Orofacial Pain, Orofacial Appearance and Psychosocial Impact. The Oral Health Impact Profile (OHIP) is the most used OHRQoL instrument designed for adults; nevertheless, it is used off‐label for children as well. Our aim was to describe the OHRQoL impact on children measured by OHIP and map the information to the 4‐dimensions framework of OHRQoL. A systematic literature review following the PRISMA statement was conducted to include studies assessing OHRQoL of children ≤ 18 years using OHIP. The OHIP seven‐domain information was converted to the OHRQoL 4‐dimension scores accompanied by their means and 95% confidence interval. Risk of bias was assessed using a six‐item modified version of quality assessment tool for prevalence studies. We identified 647 articles, after abstracts screening, 111 articles were reviewed in full text. Twelve articles were included, and their information was mapped to the 4‐dimensional OHRQoL. Most included studies had low risk of bias. OHRQoL highest impact was observed for Oral Function, Orofacial Pain, and Orofacial Appearance for children with: Decayed‐Missing‐Filled‐Surface (DMFS) of ≥10, anterior tooth extraction without replacement and untreated fractured anterior teeth, respectively. Across all oral health conditions, Psychosocial Impact was less affected than the other three dimensions. OHIP has been applied to a considerable number of children and adolescents within the literature. One instrument and a standardised set of 4‐OHRQoL dimensions across the entire lifespan seem to be a promising measurement approach in dental and oral medicine.

## 1 INTRODUCTION

Recently, there has been a paradigm change in health care indicating a shift from a biomedical perspective to a more comprehensive and broader biopsychosocial model of health.^1^, ^2^ Moreover, according to the World Health Organization (WHO), health is the state of complete physical, mental and social well‐being.^3^ Consequently, patient‐reported outcomes (PROs) and patient‐reported outcomes measures (PROMs) have emerged and are used for representing the perspective of patients regarding their health outcomes.^4^, ^5^ PROMs have a unique function in health care because they capture concepts that are only known to the patient, such as the impact of the disease in daily life, patient´s sufferings, including mental and social health aspects as well as the influence of contextual factors.^6^

In dental and oral medicine, the patient perspective is mainly captured by dental patient‐reported outcomes (dPROs) and its corresponding measures, the dental PROMs.^7^ Evidence about their importance in research as well as clinical practice is increasing,^8^ including their essential role in value‐based oral health care for implementing simultaneously economic efficiency and the optimum quality of care and value for patients.^9^, ^10^

One of the most important dPROs is oral health‐related quality of life (OHRQoL), which is commonly defined as how patients rate their well‐being and satisfaction with the current state of oral health and its psychosocial consequences.^11^ In addition, it has the potential to evaluate dental interventions from the perspective of patients in clinical practice and research.^8^

Children and adolescents can be affected by numerous oral and orofacial disorders, which impact on physical functioning and psychosocial well‐being.^11^, ^12^ Specific issues could arise when measuring OHRQoL in these age groups due to their phase of physical, cognitive, emotional, social and language development, as oral health and health cognition are considered age‐dependent.^13^, ^14^

Several instruments have been specifically developed for children and adolescents. The most often used ones includes the Child Perceptions Questionnaire (CPQ),^11^, ^15^ the Child Oral Health Impact Profile (COHIP),^16^ the Child Oral Impacts on Daily Performances (C‐OIDP)^12^ and the Early Childhood Oral Health Impact Scale (ECOHIS).^17^

In adults, the Oral Health Impact Profile (OHIP)^18^, ^19^ is the most comprehensive and widely accepted OHRQoL instrument internationally. It has sound psychometric properties and was adapted to many cultural settings.^20^ Interestingly, while OHIP was developed for adults, it is currently being applied to evaluate OHRQoL in children and adolescents in many countries.^21^, ^22^ Moreover, it was tested for its validity and reliability when applied to children and was found satisfactory.^23^

Recent studies have demonstrated that OHRQoL has four main components, so called dimensions—Oral Function, Orofacial Pain, Orofacial Appearance and Psychosocial Impact, which could provide a standardised and an efficient approach to measure what matters to patients.^7^, ^24^, ^25^, ^26^ Moreover, studies’ findings indicate that dPROMs including OHIP with this 4‐dimensional approach characterise and summarise how adults´ comprising patients and general population individuals are impacted by oral diseases.^26^, ^27^, ^28^, ^29^

Although instruments measuring OHRQoL in adults such as OHIP were also applied to measure oral health problems in children and adolescents, possibly under the assumption that capturing oral problems could be similar for both,^30^, ^31^ and were even tested for its psychometric properties and found to be suitable when applied to children,^23^ to date, a 4‐dimensional approach to describe and characterise impact across oral diseases for children has not been performed yet.

Advantages of the novel approach of characterising the oral impact for children and adults using a 4‐dimensional impact would be to allow measuring oral health impact across the entire lifespan. Adults and children could have one measurement system and not two—the current situation.

Therefore, the overall aim of this project was to perform a systematic review in order to identify publications with information about OHRQoL dimensions in children measured using OHIP and map the available seven‐domain information to the 4‐dimensional framework of OHRQoL (Oral Function, Orofacial Pain, Orofacial Appearance and Psychosocial Impact), which was characterised previously in adults. Moreover, we aimed to describe the levels of OHRQoL of different oral conditions including their clinical relevance in paediatric patients and the general population of children/adolescents.

## 2 METHODS

### 2.1 Design

A systematic literature review with the subsequent identification of instruments and extraction of data was conducted. The protocol was published in the PROSPERO database (registration number: CRD42017064033), and the PRISMA statement for reporting systematic reviews was followed.^32^

### 2.2 Inclusion criteria

1. Publications reporting about children population or paediatric patients (≤ 18 years)
2. Publications reporting OHRQoL using OHIP instrument (OHIP‐49, OHIP‐20, OHIP‐19 and OHIP‐14).^18^, ^19^, ^33^, ^34^
3. OHIP seven domains have to be available, including *functional limitations, physical pain, psychological discomfort, physical disability, psychological disability, social disability and handicap*.
4. Measure of central tendency, in addition to measure of dispersion, has to be available for the scores of the seven OHIP domains.
5. In case of mixed population of children and adults, the following criteria were adopted (mean/median age should be ≤18, or more than 50% are children ≤ 18 years, or the scores of OHIP domains have to be available for the children separately in case of mixed‐age population).
6. English language.

### 2.3 Exclusion criteria

1. Not full‐text publications (abstracts, editorials, etc).
2. Responses to the instrument are not in the proper 0 to 4 OHIP item response format.

### 2.4 Literature search process

As this review was part of a larger project on the 4 dimension‐model of the OHIP, a pool search was carried out that provided data for all four OHIP domains complemented by a manual search for the specific domains. The electronic literature search was conducted by a trained librarian (NTM, see acknowledgement) using the search terms ‘Oral Health Impact Profile’ OR ‘OHIP’ to identify articles that measure OHRQoL by OHIP for any oral health condition. The search was then limited to children or adolescents using controlled vocabulary and keywords: adolescent[MeSH] OR child[MeSH] OR child[tiab] OR adolescen*[tiab] OR teen*[tiab] OR student*[tiab]. An electronic search was conducted in MEDLINE (PubMed), EMBASE, Cochrane, CINAHL and PsycINFO from the inception of respective databases to 9 January 2019 (Table S1).

### 2.5 Screening and selection procedure

Two researchers (MO, MTJ, see acknowledgment, or KB) independently screened all titles and abstracts for inclusion based on the inclusion criteria mentioned above (criteria 1, 2 and 6 were used for abstracts and titles only). The two reviewers then discussed points of disagreements in inclusion of articles and agreed to the final decision. All potentially eligible articles underwent a full‐text review to ensure that they fulfilled the inclusion criteria. Again, disagreements within this step of the review were discussed until consensus was achieved between the two reviewers.

### 2.6 Assessment of risk of bias in included studies

Two researchers (MO and MTJ) assessed the risk of bias for the eligible articles using a modified version of the quality assessment for prevalence studies tool developed by Munn et al^35^ Only six of the ten items (item number 1, 2, 4, 5, 6 and 7) in the appraisal tool were applicable, details about the items of the assessment tool and how they were used are depicted in Table S2. Each of these six questions could be answered with ‘yes’, representing a low risk of bias, ‘unclear’ representing unknown risk or ‘no’, representing a high risk of bias. Any disagreements were resolved by arbitration.

### 2.7 Data extraction

Data extraction of the included articles was carried out by one researcher (MO), and 10% of the extracted data was checked by another researcher (MTJ). For studies that included several follow‐up time points, only the first time point was used. A particular publication could contain populations with one or more samples of patients. For each article, the following information was extracted:

- Surname of the first author of the article
- Year of publication
- Country of study
- Study design
- OHIP version used in the article
- Population characteristics
- Condition/s description of the individual samples,
- Number of the extracted subjects of the individual samples
- OHIP domain score central tendency values (eg mean) and
- OHIP domain score dispersion values (eg standard deviations).

### 2.8 Data analysis

The study determined the number of publications, the number of clinically relevant patient groups, (referred to as ‘populations’), and the number of patient samples per population with 4‐dimensional OHRQoL information.

The study also included mean values derived from any version of the OHIP questionnaire (14‐item and 49‐item). The data from OHIP‐49 were converted to OHIP‐14 means together with 95% confidence interval values restricted to fit on a 0 to 8 scale. Mean and standard deviation values were derived directly from the articles.

## 3 RESULTS

**Figure 1:**
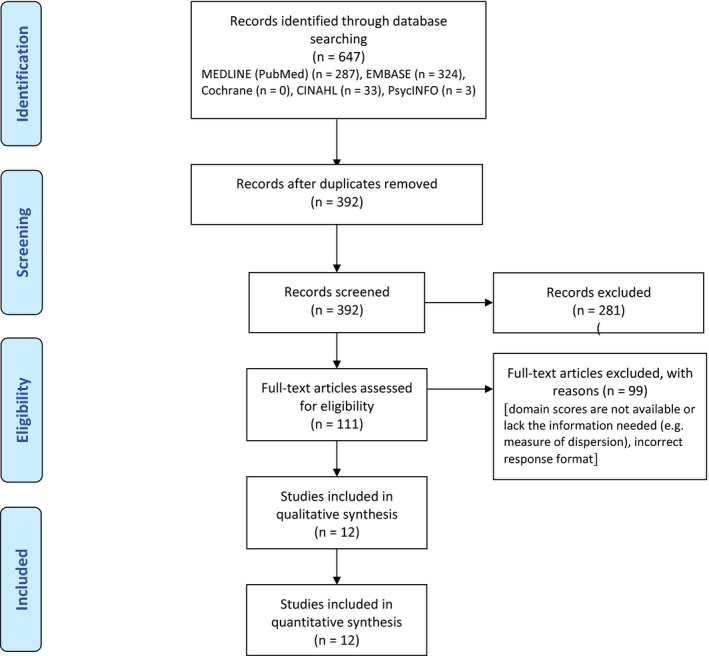
PRISMA flow diagram for the inclusion process of publications

The literature search identified 647 articles. After screening of abstracts, 111 articles were reviewed in full text (Table S3) and 12 articles met the inclusion criteria (Figure 1).

In total, 12 articles were included (nine non–follow‐up studies and the baseline of three longitudinal follow‐up studies). For our study, a total of 2687 subjects were included, and populations were identified with an overall number of 26 samples, which presented 4‐dimensional OHRQoL information. The included samples were from both the general (non‐patient) population as well as patient populations. In respect of the main conditions of the articles (*not mutually exclusive)*, 6 articles addressed malocclusion and patients undergoing orthodontic treatment. Two articles investigated skeletal discrepancies and orofacial clefting, and one investigated non‐syndromic hypodontia. Half of all articles (N = 6) assessed OHRQoL in non‐patient populations, mostly school children/adolescents and addressed several oral health conditions, for example anterior tooth extraction without replacement, presence of dental restorations, untreated fractured anterior teeth and DMFS (Table 1).

**Table 1:** Studies included in the systematic review (n = 12) for OHIP administration in children

Study	Country	Study design	OHIP type	Population (N)	Individual samples condition	Number of extracted subjects
Antoun et al,^56^ 2017	New Zealand	Matched case‐control	14	Orthodontic patients (160)	Hyperdivergent facial type	80
					Normodivergent facial type	80
Antoun et al,^57^ 2015	New Zealand	Prospective longitudinal (pre‐treatment and post‐treatment)	14	Patients undergoing orthodontic treatment (83)	Severe malocclusion (dental aesthetic index [DAI] score > 32)	30
					Orofacial clefting	24
					Severe skeletal discrepancies (require both orthodontic treatment and orthognathic Surgery)	29
Anweigi et al,^58^ 2013	Ireland	Cross‐sectional	49	Hypodontia patients (40)	Non‐syndromic Hypodontia	40
Broder et al,^59^ 2000	USA	Cross‐sectional	49	General adolescents (76)	Low DMFS (0 ‐ 5)	30
					Moderate DMFS (6 −10)	23
					High DMFS (> 10)	23
Choi et al,^60^ 2016	Korea	Cross‐sectional	14	Orthodontic patients from Orthodontic dental hospital and private clinic (214)	Malocclusion	214
De Paula et al,^61^ 2009	Brasil	Cross‐sectional	14	Public school adolescents (301)	No specific condition	301
Montero et al,^22^ 2018	Portugal	Cross‐sectional	49	Public school adolescents (782)	No specific condition	782
Nichols et al,^62^ 2018	New Zealand	Prospective longitudinal (pre‐treatment, post‐treatment, and 5 years post‐treatment)	14	Patients undergoing orthodontic treatment (57)	Severe malocclusion (dental aesthetic index [DAI] score > 32)	16
					Orofacial clefting	19
					Severe skeletal discrepancies (require both orthodontic treatment and orthognathic Surgery)	22
Oziegbe et al,^63^ 2012	Nigeria	Cross‐sectional	49	Public & private school adolescents (197)	Anterior tooth extraction without replacement	12
					Presence of dental restorations	18
					Untreated fractured anterior teeth	33
					Malocclusion	42
					Presence of orthodontic braces	25
					Controls (Free from specified dental conditions)	67
Papaioannou et al,^64^ 2011	Greece	Cross‐sectional	14	General adolescents living in urban or rural areas (515)	General adolescents living in urban	373
					General adolescents living in rural	142
Roumani et al,^65^ 2010	Greece	Cross‐sectional (Validation process)	14	General adolescents population (112)	No specific condition	112
Zhou et al,^66^ 2014	China	Prospective longitudinal (1 week, 1 month, 3 month, 6 months, and after treatment)	14	Patients undergoing orthodontic treatment (150)	Orthodontic patients—with conventional brackets	75
					Orthodontic patients—with self‐ligating brackets	75

### 3.1 Assessment of study quality

**Figure 2:**
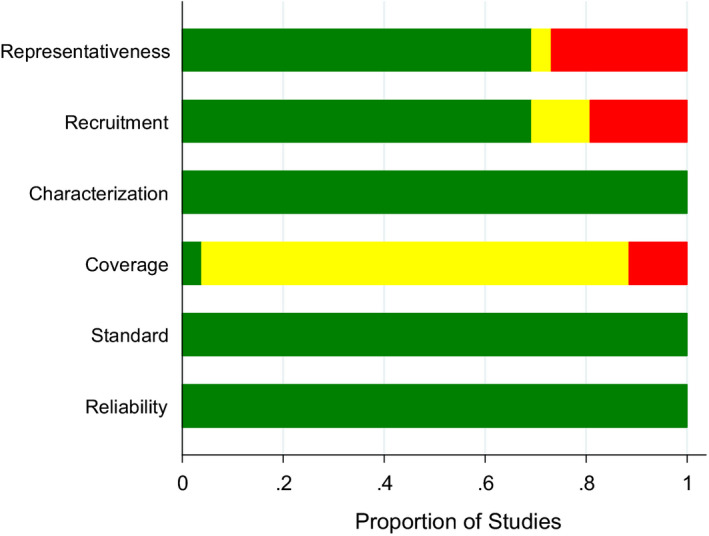
Risk of bias assessment of included studies (n = 12) presented as proportions for the assessed domains (Representativeness, Recruitment, Characterisation, Coverage, Standard and Reliability) with low (green), unclear (yellow) and high (red) risk of bias

The risk of bias assessment showed a high risk in the representativeness of the target population (does the source of population adequately represent the target population?), followed by the proper recruitment of the study participants (sampling technique, eg probability vs non‐probability samples). Another high risk and a large number of unclear risk of bias were shown by the coverage domain, which is a comparison of actual subjects/patients with the intended or planned subjects/patients (with the response rate being an important measure to assess the numerical discrepancy between both numbers). Nevertheless, because the included studies used OHIP which is validated in many languages, and well tested for its psychometric properties, the reliability and standard domains presented only low risk of bias. In addition, characterisation (if the study subjects and setting were sufficiently described) showed a low risk of bias as well. In general, a high proportion of studies (n = 8) and individual samples (n = 19) was deemed to have a low risk of bias (Figure 2 and Table S4).

### 3.2 Functional, pain, aesthetical and psychosocial OHRQoL impact

Overall results of the 0 to 8 converted OHIP scale showed that the most affected dimensions of the examined population were Oral Function, Orofacial Pain and Orofacial Appearance, while Psychosocial Impact was least affected.

**Figure 3:**
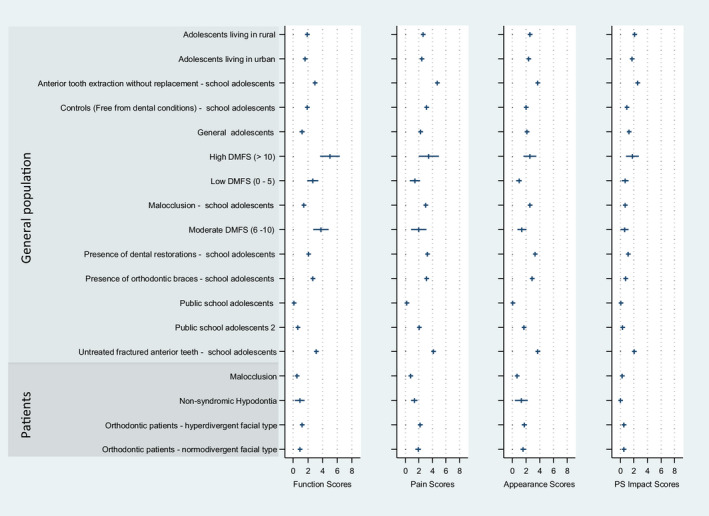
Non–follow‐up studies (n = 9) including oral conditions mapped using the mean and 95% confidence interval of the OHIP seven domains into the newly proposed 4 dimensions of OHRQoL. Conditions reported from the included articles are depicted in Table 1

When dimensions were investigated within the non–follow‐up studies (Figure 3), the Oral Function impact scores ranged between 0 and 5 on the OHIP scale and showed its highest value for the general adolescents with DMFS of ≥10. The Orofacial Pain impact score ranged also from 0 to 5 and indicated anterior tooth extraction without replacement as the highest value. Moreover, Orofacial Appearance impact scores were ranging between 0 and 4 on the OHIP scale, with anterior tooth extraction without replacement and untreated fractured anterior teeth as the highest values among the other conditions.

The Psychosocial Impact was less affected when compared to other impact dimensions. Psychosocial impact conditions were ranging from 0 to 2, with the highest values similar to the Orofacial Appearance impact in the anterior tooth extraction without replacement and untreated fractured anterior teeth.

**Figure 4:**
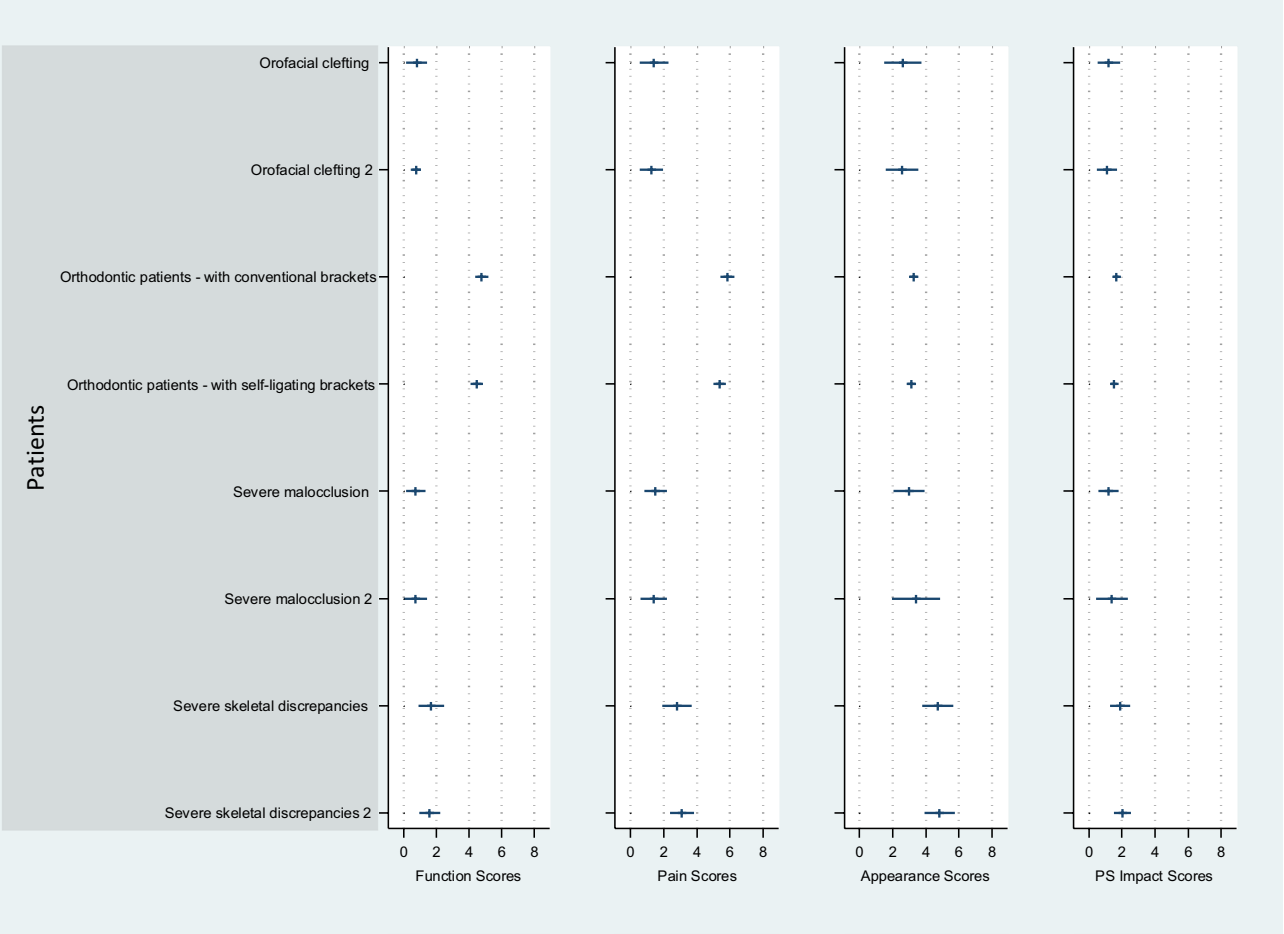
Follow‐up studies (n = 3) including oral conditions mapped using the mean and 95% confidence interval of the OHIP seven domains into the newly proposed 4 dimensions of OHRQoL. Conditions reported from the included articles are depicted in Table 1

Regarding the prospective longitudinal studies or follow‐up studies (Figure 4), the orthodontic patients with conventional brackets followed by the patients with self‐ligating brackets showed the highest impact value on the OHIP scale within the Oral Function as well as the Orofacial Pain dimensions, ranging from 0‐5 and 0‐6, respectively. However, severe skeletal discrepancies were found to be the highest score for the Orofacial Appearance impact. Similar to the non–follow‐up studies, the longitudinal studies demonstrated also in general less Psychosocial Impact compared with the three other dimensions, ranging from 0 to 2.

## 4 DISCUSSION

Oral health conditions in children are in principle similar to adults, especially in terms of how they affect their lives and quality of life. Similar to using a pharmaceutical drug off‐label for an unapproved indication or in an unapproved age group, the OHIP was used for different age groups for which it was originally developed in a considerable number of studies (n = 111). Out of those 111 articles, 12 publications met our inclusion criteria including cross‐sectional and longitudinal studies with a total of 2687 subjects suffering from several health conditions. When characterising the suffering from oral diseases of paediatric patients or non‐patient's populations, our results provided information about oral health conditions in children for which adults would suffer as well. Similar to adults, OHRQoL in children could be divided into the 4 dimensions including Oral Function, Orofacial Pain, Orofacial Appearance and Psychosocial Impact.

We investigated the non–follow‐up studies and found the highest levels of functional impairment in the general population of adolescents with DMFS of ≥10. This is consistent with adults and even elderly population studies.^36^ Here, both caries and tooth loss were strongly associated with OHRQoL, providing evidence that these conditions affect directly the oral function of individuals regardless of their age. For all 4 dimensions, when DMFS increased ranging from low to high, the impact on OHRQoL increased as well. In concordance, such an association exists also in adult populations.^37^

Regarding Orofacial Pain, interestingly, the highest pain value was found in the sample with the condition of anterior tooth extraction without replacement. The descriptive nature of our study cannot generate a reason for that, however, extraction of anterior teeth in school children could be related to possible traumatic injuries or dental caries.^38^ Moreover, the potential high levels of anxiety and psychological distress of such condition among school children could be a reason for oral psychosomatic problems.^39^ In general, there is evidence associating tooth loss in particular anterior teeth with OHRQoL impairment.^40^, ^41^

The highest values were observed within the psychosocial impact were in two conditions equally, including anterior tooth extraction without replacement and untreated fractured anterior teeth. Untreated fractured anterior teeth were emphasised by Cortes et al as having a high socio‐dental impact on daily living of children when compared to children with no traumatic dental injury.^42^ In addition, our findings indicated those same two conditions as the highest values within the appearance dimension.

Furthermore, this review included several conditions and populations, among them also populations of general adolescents from urban and rural areas. Our results showed that urban general adolescents were better in all of the four OHRQoL dimensions than the rural ones. This finding is in line with studies which investigated OHRQoL in urban and rural adults. It was found that individuals in urban area had better OHRQoL.^43^

In respect of longitudinal follow‐up studies findings, patient populations were reported, and conditions included were malocclusion, orofacial clefting, severe skeletal discrepancies and orthodontic patients with different types of brackets. Patients with severe dentofacial deformities have major conditions which affect OHRQoL in general and Orofacial Appearance in particular. Our study showed that severe skeletal discrepancies (which need both orthodontic treatment and surgery) as the highest value within the appearance dimension in the follow‐up studies. This result is in concordance with other studies which found that the satisfaction of patients with the surgery outcomes depends mainly on the improvement of aesthetics regardless of other post‐operative problems such as functional ones.^44^ Moreover, this is also consistent with adult studies which showed that adults suffering from the same condition were affected in terms of their quality of life in particular the appearance dimension.^45^ On the other hand, orthodontic patients with different types of brackets including conventional and self‐ligating ones were the highest value in pain and function impacts, and equally high as the severe skeletal discrepancies in the psychosocial impact. This finding is due to the fact that we have used the baseline data of the orthodontic patient which was one week after the placement of the brackets. OHRQoL was reported in the literature to be low at the beginning of orthodontic treatment because of many related problems in applying this new object and experiencing new feelings and discomfort including psychological ones. A study by Chen et al reported that one week after the insertion of fixed appliances, the QoL was at the worst point because the combination of physical pain, psychological discomfort and physical disability was at its highest level.^46^ Another study for older population emphasised the importance of providing information to the orthodontics patients about the temporary deterioration of OHRQoL and what they could be experiencing in the first stages of the orthodontic treatment.^47^

In both follow‐up and non–follow‐up studies, the psychosocial impact of the examined populations/conditions showed in general numerically lower values, that is better OHRQoL, of the examined conditions within the 0 to 8 converted OHIP scale when compared to the other three OHRQoL dimension impacts.

Our study included patients as well as general population studies, which was important to review the OHRQoL impact in children on both groups representing the community. This emphasises the role of OHRQoL in clinical practice including measures for personalised treatment and monitoring of patient's improvement. Additionally, OHRQoL is important for public health and community dentistry fields and can be used in epidemiological studies to determine several population characteristics and other population‐based preventive and treatment strategies. Therefore, this review demonstrated the use and applicability of the newly proposed 4‐dimension OHRQoL in patients and the general population of children.

A widely used instrument which was specifically designed for children is the child perception questionnaire. CPQ was originally developed in Canada by Jokovic et al to measure OHRQoL in children and adolescents aged from 6 to 14 years old.^11^, ^15^, ^48^ The questionnaire consists of four domains including: oral symptoms, functional restrictions, emotional impairment and social impairment. The CPQ domains are also consistent with the newly proposed four impacts because the socio‐emotional dimension represents the Psychosocial Impact and the Orofacial Appearance, while the symptoms‐functioning dimension represents mainly Oral Function and Orofacial Pain.^49^ A recent systematic review by Ferrando‐Magraner et al compared OHIP and CPQ in regard to malocclusion and showed similar results in the administration of both instruments in children. Both instruments reported a significant improvement in OHRQoL at the end of orthodontic treatment in children.^50^ Another study by Oscarson et al^51^ investigated using OHIP‐14 and CPQ 11‐14 in a population of 19 years old and assumed that in principle there are similarities between the two instruments and that the CPQ 11‐14 would be applicable for the 19‐year‐olds to understand and complete. These studies investigated the association between OHIP and OHRQoL instruments which were made specifically for children. It provided evidence about the results of OHIP when administered to children. Therefore, OHIP would most likely be similar to other paediatric OHRQoL instruments. Moreover, OHIP ‘behaves’ like a genuine instrument for children because it measures the four dimensions which are measured by all other instruments. For adults, it could be shown that the four OHRQoL dimensions are the major attribute that underlie not only OHRQoL instruments but also all generic dPROMs.^52^ Similar findings may also be expected for children.

Prevention strategies and proper monitoring of oral health throughout life lead to preservation of good OHRQoL. Consequently, OHRQoL assessment is an integral component in any treatment or intervention strategy and should be performed prior to any preventative or therapeutic intervention. ‘One instrument for all ages’ to measure oral health impact would be an approach which will allow testing and investigating several hypotheses related to live‐long impact of oral diseases. A good example for that is malocclusion. With a proper measurement system in place, the long‐term impact, that is when patients are adults, of malocclusion and the effects of orthodontic treatments could be investigated.

Another example for a live‐long impact is caries, starting from early childhood caries (ECC), to caries affecting individuals later in life and impacting all aspects of OHRQOL. Previous caries experience is considered the most dominant caries predictor in all age groups.^53^, ^54^ Consequently, caries and its impact on OHRQoL could be investigated longitudinally to include wider age ranges.

Therefore, the concept of 4‐dimensions’ impact of OHRQoL when used for adults and children could allow measuring patients´ suffering across all ages, as well as providing more consistent and standardised dimensions of the OHRQoL. Accordingly, a proper and continuous monitoring strategy for preserving oral health of populations and implementing value‐based health care will be achieved. Furthermore, it would facilitate research projects for comparing different age groups using a standardised instrument.

Regarding younger children assessment, since the 4 dimensions are the major areas of dental patients' suffering across the entire age span, methods to collect the information, for example self‐ versus proxy assessment or the mode of questionnaire administration, can be flexibly adapted according to the specific settings and the needs of the examined persons. However, more research will be needed to investigate this approach in younger children.

In general, standardisation and consistency of the measurements used for a specific construct, for example OHRQoL, are important for comparing different studies, populations and different age groups as well as enabling the follow‐up of persons over the life span.^55^ In this study, we are presenting evidence that OHIP can likely be successfully applied to older children and their respective conditions. Consequently, it supports with empirical data the concept of having a ‘one instrument’ measuring the main four dimensions of OHRQoL for all age groups. While such an approach is promising, more methodological work towards the goal of a unified impact measurement over the entire life span is necessary. For example, such a universal metric for children and adults can be further psychometrically investigated and improved by using modern psychometric approaches such as examining fit to the Rasch model. Moreover, whether OHRQoL items function differently for children and adults could be studied by examining the differential item functioning (DIF). Such techniques are essential to provide psychometrically solid dental patient‐reported outcome measures.

### 4.1 Limitation

Through OHIP being used ‘off‐label’, that is applying the instrument to an age groups it was not designed for, standardised 4‐dimensional information became available and the represented oral health conditions was low. Interestingly, researchers used OHIP in children and found it suitable for measuring several conditions including such important dental patient populations such as patients with malocclusion. Although many OHIP papers exist in children, we could not use all of them for the current project because we needed domain values (for the dimensions). Therefore, more OHRQoL information is actually available.

## 5 CONCLUSION

A considerable number of international researchers have applied OHIP, an OHRQoL instrument for adults, to children and adolescents. Moreover, using OHIP for paediatric patients and the general population of children and mapping the information to the four OHRQoL dimensions Oral Function, Orofacial Pain, Orofacial Appearance and Psychosocial Impact provide meaningful results about magnitude and variation of OHRQoL impairment. While more research on the psychometric properties of OHIP in children is needed, one instrument and a standardised set of four OHRQoL dimensions across the entire lifespan seems to be a promising measurement approach in dental and oral medicine.

## CONFLICT OF INTEREST

The authors declare that no conflicts of interest exist.

## AUTHORS CONTRIBUTIONS

MO, KB and TS conceived the ideas. MO and KB collected the data. MO, KB and TS analysed the data. MO, KB and TS wrote the manuscript. MO, KB and TS revised and approved the manuscript.

### Peer Review

The peer review history for this article is available at https://publons.com/publon/10.1111/joor.13066.

## Supporting information

Supplementary Material
