# Supplementary material for: Four‐dimensional oral health‐related quality of life impact in children: A systematic review
Source: J Oral Rehabil. 2020 Aug 19;48(3):293–304. doi: 10.1111/joor.13066 (PMC7984176; doi:10.1111/joor.13066)
Supplement: Supplementary file 1 — Supplementary Material [file JOOR-48-293-s001.docx]

**Supplementary table 1:** Search Strategies adopted for the systematic review.

| **MEDLINE (PubMed)** |
| --- |
| (exp adolescent/ OR exp child/ OR child.ab, ti. OR adolescen*.ab,ti. OR teen*.ab, ti. OR student*.ab, ti.)  ***NOT:***  ("ontario health insurance plan"[tiab] OR COHIP[tiab] OR "Child Oral Health Impact Profile"[tiab]) |
| **Embase** via the Ovid platform (1974 to 2019 January 08) * |
| (OHIP.ab, ti. OR "oral health impact profile".ab,ti.) AND (adolescent/ OR child/ OR child.ab, ti. OR adolescen*.ab,ti. OR teen*.ab, ti. OR student*.ab, ti.)  ***NOT:***  ("ontario health insurance plan".ab,ti. OR COHIP.ab, ti. OR "Child Oral Health Impact Profile".ab,ti.) |
| **Cinahl** via EBSCO |
| (OHIP (TI) OR OHIP (AB)) OR (“oral health impact profile” (TI) OR “oral health impact profile” (AB)) AND (MH “Child+” OR Child ((TI OR AB) OR Adolescent (TX All Text) OR Teen* (TI, AB) OR Student* (TI, AB))  ***NOT:***  "Child Oral Health Impact Profile” OR “Ontario health insurance plan” |
| **PsycINFO** via Ovid platform (1987 to December Week 5 2018) * |
| (OHIP.ab, ti. OR "oral health impact profile".ab,ti.) AND (child,ab, ti. OR adolecen*.ab, ti. OR teen*.ab, ti. OR student*.ab, ti.)  ***NOT:***  ontario health insurance plan".ab,ti. OR COHIP.ab, ti. OR “Child Oral Health Impact Profile”.ab, ti. |
| **Cochrane** via Wiley |
| (OHIP.ab, ti. OR "oral health impact profile".ab,ti.) |

N.B. All searches were limited to English and 1990 - January 9, 2019

** Embase and PsycINFO databases comprise date ranges of included published articles. The date ranges and information depicted in the table refer to all the content available in those databases at the time of the search. For these searches the articles were then limited to the ones published after 1990 till January 9, 2019.*

**Supplementary table 2:** The 10-items appraisal tool used for assessing the quality of studies included ^1^: Not applicable items were excluded from the assessment tool (highlighted in gray) and reasons for exclusion are depicted.

| **Item No.** | **Items for the assessment of risk of bias** | **Yes /NO/ Unclear/ Not applicable** |
| --- | --- | --- |
| 1. | Was the sample representative of the target population? | **Yes:** Source population adequately represents target population.  **No:** Source population does not adequately represent target population.  **Unclear:** Not enough information provided. |
| 2. | Were study participants recruited in an appropriate way? | **Yes**: Probability sample  **No**: Other than probability sample  **Unclear**: Sampling type not mentioned. |
| 3. | Was the sample size adequate? | **Not applicable:** Sample size determines the precision of the estimate. We take the studies with the precision they have. Studies sample size is always adequate for the purpose of our study. |
| 4. | Were the study subjects and the setting described in detail? | **Yes:** Sufficient information about subjects/setting and measurement of the essential subject characteristics is sufficient.  **No:** Insufficient information about subjects/setting or measurement of the essential subject characteristics is not sufficient  **Unclear:** Not enough information to decide. |
| 5. | Was the data analysis conducted with sufficient coverage of the identified sample? | **Yes:** Response rate sufficient  **Unclear:** response rate not mentioned  **No:** Response rate not sufficient. |
| 6. | Were objective and standard criteria used for the measurement of the condition? | **YES:** OHIP language-version was referenced or authors provide evidence for OHIP score validity in the paper.  **No:** OHIP language-version was not referenced and no evidence of score validity is provided.  **Unclear:** category not necessary |
| 7. | Was the condition measured reliably? | **Yes:** OHIP language-version was referenced or authors provide evidence for OHIP score reliability in the paper.  **No:** OHIP language-version was not referenced and no evidence of score reliability is provided.  **Unclear:** category not necessary |
| 8. | Was there appropriate statistical analysis? | **Not applicable:** We use measures of central tendency (e.g. mean) and measure of dispersion (e.g. standard deviation), and we have assumed they were properly calculated. |
| 9. | Are all important confounding factors/subgroups/differences identified and accounted for? | **Not applicable:** Confounding is a concept for association studies which we didn´t find as applicable here. |
| 10. | Were subpopulations identified using objective criteria? | **Not applicable:** We have considered OHIP domain values for central tendency (e.g. mean) and dispersion (e.g. standard deviation) which characterize OHRQoL impairment in any particular population. |

**Supplementary table 3:** Excluded articles which were qualified for full-text review, and reasons for exclusion.

| **No.** | **Authors** | **Year** | **Reason for exclusion** |
| --- | --- | --- | --- |
|  | S. Acharya and D. K. Sangam ^2^ | 2008 | Age of participants doesn’t meet the inclusion criteria |
|  | G. Almoznino, D. J. Aframian, Y. Sharav, Y. Sheftel, A. Mirzabaev and A. Zini ^3^ | 2015 | Age of participants doesn’t meet the inclusion criteria |
|  | G. Almoznino, A. Zini, D. J. Aframian, E. Kaufman, A. Lvovsky, A. Hadad and L. Levin ^4^ | 2015 | Age of participants doesn’t meet the inclusion criteria |
|  | G. Almoznino, A. Zini, Y. Sharav, A. Shahar, H. Zlutzky, Y. Haviv, A. Lvovsky and D. J. Aframian ^5^ | 2015 | Age of participants doesn’t meet the inclusion criteria |
|  | G. Almoznino, A. Zini, Y. Sharav, R. Yanko, A. Lvovsky and D. J. Aframian ^6^ | 2016 | Age of participants doesn’t meet the inclusion criteria |
|  | E. Alpkilic Baskirt, G. Ak and B. Zulfikar ^7^ | 2009 | Domain data not available |
|  | A. C. Amaral Loureiro, F. Oliveira Costa and J. Eustaquio da Costa ^8^ | 2007 | Domain data not available |
|  | A. N. Anosike, O. O. Sanu and O. O. da Costa ^9^ | 2010 | Domain data not available |
|  | R. P. Antoniazzi, L. S. Fischer, C. E. A. Balbinot, S. P. Antoniazzi and J. A. Skupien ^10^ | 2017 | Age of participants doesn’t meet the inclusion criteria |
|  | R. P. Antoniazzi, F. B. Zanatta, T. M. Ardenghi and C. A. Feldens ^11^ | 2018 | Age of participants doesn’t meet the inclusion criteria |
|  | L. Anweigi, P. Finbarr Allen and H. Ziada ^12^ | 2013 | Domain data not available |
|  | A. Ashari and A. M. Mohamed ^13^ | 2016 | Age of participants doesn’t meet the inclusion criteria |
|  | A. Ballon, K. Laudemann, R. Sader and C. A. Landes ^14^ | 2011 | Domain data not available |
|  | S. Banerjee, R. Banerjee, U. Shenoy, S. Agarkar and S. Bhattacharya ^15^ | 2018 | Domain data not available and wrong response format |
|  | E. Bardellini, F. Amadori and A. Majorana ^16^ | 2016 | Domain data not available |
|  | E. Bardellini, F. Amadori, J. Merlo, A. Ferri and A. Majorana | 2017 | Article not available (Abstract meeting only) |
|  | A. Barkokebas, I. H. Silva, S. C. de Andrade, A. A. Carvalho, L. A. Gueiros, S. M. Paiva and J. C. Leao ^17^ | 2015 | Domain data not available |
|  | M. Baron, M. Hudson, S. Tatibouet, R. Steele, E. Lo, S. Gravel, G. Gyger, T. El Sayegh, J. Pope, A. Fontaine, A. Masseto, D. Matthews, E. Sutton, N. Thie, N. Jones, M. Copete, D. Kolbinson, J. Markland, G. Nogueira-Filho, D. Robinson and M. Gornitsky ^18^ | 2014 | Age of participants doesn’t meet the inclusion criteria |
|  | P. Allison, D. Locker, A. Jokovic and G. Slade ^19^ | 1999 | Insufficient information |
|  | I. Alajbeg and M. Bagic ^20^ | 2010 | Article not available |
|  | M. A. Alghamdi, N. J. Farsi and A. H. Hassan ^21^ | 2017 | Insufficient information |
|  | M. Barros Vde, P. I. Seraidarian, M. I. Cortes and L. V. de Paula ^22^ | 2009 | Article not available |
|  | R. S. Bastos, E. S. Carvalho, A. Xavier, M. L. Caldana, J. R. Bastos and J. R. Lauris ^23^ | 2012 | Domain data not available |
|  | E. Bernabe, C. M. de Oliveira and A. Sheiham ^24^ | 2008 | Domain data not available |
|  | E. Bernabe, C. M. de Oliveira, A. Sheiham and G. Tsakos ^25^ | 2009 | Domain data not available |
|  | L. M. Bezinelli, F. P. Eduardo, V. D. Neves, L. Correa, R. M. Lopes, E. Michel-Crosato, N. Hamerschlak and M. G. Biazevic ^26^ | 2016 | Age of participants doesn’t meet the inclusion criteria |
|  | M. G. Biazevic, R. R. Rissotto, E. Michel-Crosato, L. A. Mendes and M. O. Mendes ^27^ | 2008 | Domain data not available |
|  | S. Bilic, I. Blomberg, K. Burry, E. Chong, E. Yeung and A. Ariyawardana ^28^ | 2017 | Age of participants doesn’t meet the inclusion criteria |
|  | A. Blanco-Aguilera, A. Blanco-Hungria, L. Biedma-Velazquez, R. Serrano-Del-Rosal, L. Gonzalez-Lopez, E. Blanco-Aguilera and R. Segura-Saint-Gerons ^29^ | 2014 | Age of participants doesn’t meet the inclusion criteria |
|  | M. C. Bortoluzzi, R. Manfro, I. C. Soares and A. A. Presta ^30^ | 2011 | Insufficient information |
|  | M. A. Bozzella, L. J. Motta, T. A. Alfaya, C. V. D. Gouvea and S. K. Bussadori ^31^ | 2013 | Wrong response format |
|  | D. S. Brennan and A. J. Spencer ^32^ | 2005 | Article not available |
|  | D. S. Brennan and A. J. Spencer ^33^ | 2014 | Domain data not available |
|  | J. R. Broughton, J. Teh Maipi, M. Person, A. Randall and W. M. Thomson ^34^ | 2012 | Domain data not available |
|  | I. M. Busato, S. A. Ignacio, J. A. Brancher, A. M. Gregio, M. A. Machado and L. R. Azevedo-Alanis ^35^ | 2009 | Domain data not available |
|  | I. M. Busato, S. A. Ignacio, J. A. Brancher, S. T. Moyses and L. R. Azevedo-Alanis ^36^ | 2012 | Domain data not available |
|  | I. M. Busato, M. Thomaz, A. I. Toda, D. G. Alanis, B. H. Franca, A. A. de Lima and L. R. Azevedo-Alanis ^37^ | 2013 | Age of participants doesn’t meet the inclusion criteria |
|  | F. Caglayan, O. Altun, O. Miloglu, M. D. Kaya and A. B. Yilmaz ^38^ | 2009 | Age of participants doesn’t meet the inclusion criteria |
|  | J. C. Carvalho, H. D. Mestrinho, S. Stevens and A. J. van Wijk ^39^ | 2015 | Age of participants doesn’t meet the inclusion criteria |
|  | M. Cassetta and F. Altieri ^40^ | 2017 | Domain data not available |
|  | M. Cassetta, S. Di Carlo, M. Giansanti, V. Pompa, G. Pompa and E. Barbato ^41^ | 2012 | Insufficient information |
|  | M. Chen, D. W. Wang and L. P. Wu ^42^ | 2010 | Insufficient information |
|  | P. R. Colussi, F. N. Hugo, F. W. Muniz and C. K. Rosing ^43^ | 2017 | Domain data not available |
|  | L. A. Crocombe and G. D. Mahoney ^44^ | 2016 | Domain data not available |
|  | C. M. de Oliveira and A. Sheiham ^45^ | 2003 | Insufficient information |
|  | C. M. de Oliveira and A. Sheiham ^46^ | 2004 | Insufficient information |
|  | M. J. Fernandes, D. A. Ruta, G. R. Ogden, N. B. Pitts and S. A. Ogston ^47^ | 2006 | Age of participants doesn’t meet the inclusion criteria |
|  | D. Feu, B. H. de Oliveira, M. A. de Oliveira Almeida, H. A. Kiyak and J. A. Miguel ^48^ | 2010 | Domain data not available |
|  | D. Feu, J. A. Miguel, R. K. Celeste and B. H. Oliveira ^49^ | 2013 | Age of participants doesn’t meet the inclusion criteria |
|  | L. Giannetti, A. Murri, F. Vecci and R. Gatto ^50^ | 2007 | Domain data not available |
|  | J. G. Goelzer, O. E. Becker, O. L. Haas Junior, N. Scolari, M. F. Santos Melo, C. Heitz and R. B. de Oliveira ^51^ | 2014 | Age of participants doesn’t meet the inclusion criteria |
|  | V. Goh, D. Nihalani, K. W. S. Yeung, E. F. Corbet and W. K. Leung ^52^ | 2018 | Age of participants doesn’t meet the inclusion criteria |
|  | M. Hanisch, S. Wiemann, S. Jung, J. Kleinheinz and L. Bohner ^53^ | 2018 | Domain data not available |
|  | S. L. He and J. H. Wang ^54^ | 2015 | Age of participants doesn’t meet the inclusion criteria |
|  | L. Hongxing, T. List, I. M. Nilsson, A. Johansson and A. N. Astrom ^55^ | 2014 | Domain data not available |
|  | S. Ishida, Y. Shibuya, M. Kobayashi and T. Komori ^56^ | 2015 | Age of participants doesn’t meet the inclusion criteria |
|  | A. Jamilian, B. Kiaee, S. Sanayei, S. Khosravi and L. Perillo ^57^ | 2016 | Domain data not available |
|  | M. T. John, P. Hujoel, D. L. Miglioretti, L. LeResche, T. D. Koepsell and W. Micheelis ^58^ | 2004 | Insufficient information |
|  | S. Keles, F. Abacigil and F. Adana ^59^ | 2018 | Wrong response format |
|  | N. Kenig and J. Nikolovska ^60^ | 2012 | Age of participants doesn’t meet the inclusion criteria |
|  | N. Khalifa, P. F. Allen, N. H. Abu-bakr and M. E. Abdel-Rahman ^61^ | 2013 | Insufficient information |
|  | A. Kilinc and U. Ertas ^62^ | 2015 | Age of participants doesn’t meet the inclusion criteria |
|  | E. Kurklu-Gurleyen, M. Ogut-Erisen, O. Cakir, O. Uysal and G. Ak ^63^ | 2016 | Age of participants doesn’t meet the inclusion criteria |
|  | S. Lee, C. McGrath and N. Samman ^64^ | 2007 | Age of participants doesn’t meet the inclusion criteria |
|  | L. Levin, A. Zini, J. Levine, M. Weiss, R. A. Lev, A. Hai, D. Chebath-Taub and G. Almoznino ^65^ | 2018 | Domain data not available |
|  | B. Lewandowski, E. Szeliga, E. Czenczek-Lewandowska, D. Ozga, A. Kontek, M. Migut, G. Magon, J. Kosydar-Bochenek and A. Kuzdzal ^66^ | 2018 | Age of participants doesn’t meet the inclusion criteria |
|  | X. Li, K. Zhu, F. Liu and H. Li ^67^ | 2014 | Wrong response format |
|  | L. J. Liu, W. Xiao, Q. B. He and W. W. Jiang ^68^ | 2012 | Age of participants doesn’t meet the inclusion criteria |
|  | Z. Liu, C. McGrath and U. Hagg ^69^ | 2011 | Age of participants doesn’t meet the inclusion criteria |
|  | R. Lopez and V. Baelum ^70^ | 2006 | Wrong response format |
|  | R. Lopez and V. Baelum ^71^ | 2007 | Domain data not available |
|  | C. V. R. Maia, F. M. Mendes and D. Normando ^72^ | 2018 | Wrong response format |
|  | C. M. Manjith, S. K. Karnam, S. Manglam, M. N. Praveen and A. Mathur ^73^ | 2012 | Insufficient information |
|  | A. V. Mary, J. Mahendra, J. John, J. Moses, A. V. R. Ebenezar and R. Kesavan ^74^ | 2017 | Insufficient information |
|  | M. Masood, Y. Masood and T. Newton ^75^ | 2014 | Age of participants doesn’t meet the inclusion criteria |
|  | M. Masood, Y. Masood, T. Newton and S. Lahti ^76^ | 2015 | Age of participants doesn’t meet the inclusion criteria |
|  | O. A. Obilade, O. O. Sanu and O. O. Costa ^77^ | 2016 | Article not available |
|  | D. C. Oliveira, F. M. Ferreira, A. Morosini Ide, C. C. Torres-Pereira, S. Martins Paiva and F. C. Fraiz ^78^ | 2015 | Article not available |
|  | G. Pousette Lundgren, A. Karsten and G. Dahllof ^79^ | 2015 | Different domains |
|  | V. Ravaghi, M. M. Ardakan, S. Shahriari, N. Mokhtari and M. Underwood ^80^ | 2011 | Domain data not available |
|  | J. Rimal and A. Shrestha ^81^ | 2015 | Age of participants doesn’t meet the inclusion criteria |
|  | F. Sampogna, B. Soderfeldt, B. Axtelius, F. Bergamo, P. Gisondi, C. Di Pietro, L. Alessandroni, C. Pagliarello, G. Zino, P. Pallotta, S. Tabolli and D. Abeni ^82^ | 2011 | Age of participants doesn’t meet the inclusion criteria |
|  | T. T. Santa-Rosa, R. C. Ferreira, A. M. Drummond, C. S. De Magalhaes, A. M. Vargas and E. F. E. Ferreira ^83^ | 2014 | Domain data not available |
|  | R. Saub, D. Locker and P. Allison ^84^ | 2008 | Age of participants doesn’t meet the inclusion criteria |
|  | A. Schmidt, R. Ciesielski, W. Orthuber and B. Koos ^85^ | 2013 | Age of participants doesn’t meet the inclusion criteria |
|  | S. Siluvai, N. Kshetrimayum, C. V. Reddy, S. Siddanna, M. Manjunath and S. Rudraswamy ^86^ | 2015 | Domain data not available |
|  | I. Silva, C. Cardemil, H. Kashani, F. Bazargani, P. Tarnow, L. Rasmusson and F. Suska ^87^ | 2016 | Age of participants doesn’t meet the inclusion criteria |
|  | L. F. Silva, E. B. Thomaz, H. V. Freitas, A. L. Pereira, C. C. Ribeiro and C. M. Alves ^88^ | 2016 | Domain data not available |
|  | M. F. Silveira, J. P. Maroco, R. S. Freire, A. M. Martins and L. F. Marcopito ^89^ | 2014 | Wrong response format |
|  | K. K. Soe, S. Gelbier and P. G. Robinson ^90^ | 2004 | Domain data not available |
|  | K. M. Stange, R. Lindsten and K. Bjerklin ^91^ | 2016 | Domain data not available |
|  | F. Stelzle, M. Rohde, N. Oetter, K. Krug, M. Riemann, W. Adler, F. W. Neukam and C. Knipfer ^92^ | 2017 | Insufficient information |
|  | H. Terheyden and F. Wusthoff ^93^ | 2015 | Domain data not available |
|  | J. E. Tinoco-Araujo, E. S. Orti-Raduan, D. Santos, V. A. Colturato, M. P. Souza, M. A. Mauad, T. C. Saggioro, R. S. Bastos and P. S. da Silva Santos ^94^ | 2015 | Age of participants doesn’t meet the inclusion criteria |
|  | K. M. Van Lierde, A. Luyten, E. D'Haeseleer, G. Van Maele, L. Becue, E. Fonteyne, P. Corthals and G. De Pauw ^95^ | 2015 | Domain data not available |
|  | D. Yu, F. Wang, X. Wang, B. Fang and S. G. Shen ^96^ | 2013 | Article not available |
|  | P. L. Yule, J. Durham, H. Playford, M. A. Moufti, J. Steele, N. Steen, R. W. Wassell and R. Ohrbach ^97^ | 2015 | Age of participants doesn’t meet the inclusion criteria |
|  | F. B. Zanatta, T. M. Ardenghi, R. P. Antoniazzi, T. M. Pinto and C. K. Rosing ^98^ | 2012 | Age of participants doesn’t meet the inclusion criteria |
|  | D. H. Zheng, X. X. Wang, Y. R. Su, S. Y. Zhao, C. Xu, C. Kong and J. Zhang ^99^ | 2015 | Age of participants doesn’t meet the inclusion criteria |

**Supplementary table 4**: The detailed quality assessment for each single study.

| **Study** | **Representative**  **(Q1)** | **Recruitment**  **(Q2)** | **Characterization**  **(Q4)** | | **Coverage**  **(Q5)** | **Standard**  **(Q6)** | **Reliability**  **(Q7)** |
| --- | --- | --- | --- | --- | --- | --- | --- |
| Antoun et al.^100^ , 2017 | N | U | Y | | U | Y | Y |
| Antoun et al.^101^ , 2015 | Y | Y | Y | U | | Y | Y |
| Anweigi et al.^102^ , 2013 | U | U | Y | U | | Y | Y |
| Broder et al.^103^ , 2000 | N | N | Y | U | | Y | Y |
| Choi et al.^104^ , 2016 | N | N | Y | U | | Y | Y |
| De Paula et al.^105^ , 2009 | N | N | Y | U | | Y | Y |
| Montero et al.^106^ , 2018 | Y | Y | Y | Y | | Y | Y |
| Nichols et al.^107^ , 2018 | Y | Y | Y | N | | Y | Y |
| Oziegbe et al.^108^ , 2012 | Y | Y | Y | U | | Y | Y |
| Papaioannou et al.^109^ , 2011 | Y | Y | Y | U | | Y | Y |
| Roumani et al.^110^ , 2010 | Y | Y | Y | U | | Y | Y |
| Zhou et al.^111^, 2014 | Y | Y | Y | U | | Y | Y |

N.B. Yes= Y, No= N, and Unclear= U.

**Supplementary file references**

1. Munn Z, Moola S, Riitano D, Lisy K. The development of a critical appraisal tool for use in systematic reviews addressing questions of prevalence. *International journal of health policy and management.* 2014;3(3):123-128.

2. Acharya S, Sangam D. Oral health‐related quality of life and its relationship with health locus of control among Indian dental university students. *European Journal of Dental Education.* 2008;12(4):208-212.

3. Almoznino G, Aframian D, Sharav Y, Sheftel Y, Mirzabaev A, Zini A. Lifestyle and dental attendance as predictors of oral health‐related quality of life. *Oral diseases.* 2015;21(5):659-666.

4. Almoznino G, Zini A, Aframian DJ, et al. Oral health related quality of life in young individuals with dental anxiety and exaggerated gag reflex. *Oral Health Prev Dent.* 2015;13(5):435-440.

5. Almoznino G, Zini A, Sharav Y, et al. Sleep quality in patients with dental anxiety. *Journal of psychiatric research.* 2015;61:214-222.

6. Almoznino G, Zini A, Sharav Y, Yanko R, Lvovsky A, Aframian DJ. Overlap between dental anxiety, gagging and Blood-Injection-Injury related fears—A spectrum of one multidimensional phenomenon. *Physiology & behavior.* 2016;165:231-238.

7. Alpkilic Baskirt E, Ak G, Zulfikar B. Oral and general health-related quality of life among young patients with haemophilia. *Haemophilia : the official journal of the World Federation of Hemophilia.* 2009;15(1):193-198.

8. Amaral Loureiro AC, Oliveira Costa F, Eustaquio da Costa J. The impact of periodontal disease on the quality of life of individuals with Down syndrome. *Down's syndrome, research and practice : the journal of the Sarah Duffen Centre.* 2007;12(1):50-54.

9. Anosike A, Sanu O, Da Costa O. Malocclusion and its impact on quality of life of school children in Nigeria. *West African journal of medicine.* 2010;29(6).

10. Antoniazzi RP, Fischer LdS, Balbinot CEA, Antoniazzi SP, Skupien JA. Impact of excessive gingival display on oral health‐related quality of life in a Southern Brazilian young population. *Journal of clinical periodontology.* 2017;44(10):996-1002.

11. Antoniazzi RP, Zanatta FB, Ardenghi TM, Feldens CA. The use of crack and other illicit drugs impacts oral health‐related quality of life in Brazilians. *Oral diseases.* 2018;24(3):482-488.

12. Anweigi L, Allen PF, Ziada H. Impact of resin bonded bridgework on quality of life of patients with hypodontia. *Journal of dentistry.* 2013;41(8):683-688.

13. Ashari A, Mohamed AM. Relationship of the Dental Aesthetic Index to the oral health-related quality of life. *The Angle Orthodontist.* 2016;86(2):337-342.

14. Ballon A, Laudemann K, Sader R, Landes CA. Patients' preoperative expectations and postoperative satisfaction of dysgnathic patients operated on with resorbable osteosyntheses. *Journal of Craniofacial Surgery.* 2011;22(2):730-734.

15. Banerjee S, Banerjee R, Shenoy U, Agarkar S, Bhattacharya S. Effect of orthodontic pain on quality of life of patients undergoing orthodontic treatment. *Indian Journal of Dental Research.* 2018;29(1):4.

16. Bardellini E, Amadori F, Majorana A. Oral hygiene grade and quality of life in children with chemotherapy‐related oral mucositis: a randomized study on the impact of a fluoride toothpaste with salivary enzymes, essential oils, proteins and colostrum extract versus a fluoride toothpaste without menthol. *International journal of dental hygiene.* 2016;14(4):314-319.

17. Barkokebas A, Silva IHM, de Andrade SC, et al. Impact of oral mucositis on oral‐health‐related quality of life of patients diagnosed with cancer. *Journal of Oral Pathology & Medicine.* 2015;44(9):746-751.

18. Baron M, Hudson M, Tatibouet S, et al. The Canadian systemic sclerosis oral health study: orofacial manifestations and oral health-related quality of life in systemic sclerosis compared with the general population. *Rheumatology.* 2014;53(8):1386-1394.

19. Allison P, Locker D, Jokovic A, Slade G. A cross-cultural study of oral health values. *Journal of Dental research.* 1999;78(2):643-649.

20. Alajbeg I, Bagic M. Information Leaflets Improve Oral Health Related Quality of Life in Patients with Oral Diseases: 187. *Oral Diseases.* 2010;16(6).

21. Alghamdi MA, Farsi NJ, Hassan AH. Comparison of oral health-related quality of life of patients treated by palatal expanders with patients treated by fixed orthodontic appliances. *Patient preference and adherence.* 2017;11:699.

22. Barros MV, Seraidarian PI, Côrtes M. The impact of orofacial pain on the quality of life of patients with temporomandibular disorder. *Journal of orofacial pain.* 2009;23(1):28-37.

23. Bastos RS, Carvalho ÉS, Xavier A, Caldana ML, Bastos JR, Lauris JR. Dental caries related to quality of life in two Brazilian adolescent groups: a cross‐sectional randomised study. *International dental journal.* 2012;62(3):137-143.

24. Bernabé E, de Oliveira CM, Sheiham A. Comparison of the discriminative ability of a generic and a condition-specific OHRQoL measure in adolescents with and without normative need for orthodontic treatment. *Health and quality of life outcomes.* 2008;6(1):64.

25. Bernabé E, De Oliveira CM, Sheiham A, Tsakos G. Assessing levels of agreement between two commonly used oral health‐related quality of life measures. *Journal of public health dentistry.* 2009;69(3):143-148.

26. Bezinelli LM, Eduardo F, Neves V, et al. Quality of life related to oral mucositis of patients undergoing haematopoietic stem cell transplantation and receiving specialised oral care with low‐level laser therapy: a prospective observational study. *European journal of cancer care.* 2016;25(4):668-674.

27. Biazevic MGH, Rissotto RR, Michel-Crosato E, Mendes LA, Mendes MOA. Relationship between oral health and its impact on quality of life among adolescents. *Brazilian oral research.* 2008;22(1):36-42.

28. Bilic S, Blomberg I, Burry K, Chong E, Yeung E, Ariyawardana A. Oral‐health‐related quality of life of dental patients: a hospital based study in far north Queensland, Australia. *Journal of investigative and clinical dentistry.* 2017;8(3):e12216.

29. Blanco-Aguilera A, Blanco-Hungría A, Biedma-Velázquez L, et al. Application of an oral health-related quality of life questionnaire in primary care patients with orofacial pain and temporomandibular disorders. *Medicina oral, patologia oral y cirugia bucal.* 2014;19(2):e127.

30. Bortoluzzi M-C, Manfro R, Soares I-C, Presta A-A. Cross-cultural adaptation of the orthognathic quality of life questionnaire (OQLQ) in a Brazilian sample of patients with dentofacial deformities. *Med Oral Patol Oral Cir Bucal.* 2011;16(5):e694-699.

31. Bozzella MA, Motta L, Alfaya TA, Gouvêa C, Bussadori S. Evaluation of oral health-related quality of life at public schools in São Vicente, Brazil. *Clinical and Experimental Medical Letters.* 2013;54:179-183.

32. Brennan D, Spencer A. Comparison of a generic and a specific measure of oral health related quality of life. *Community dental health.* 2005;22(1):11-18.

33. Brennan DS, Spencer AJ. Dental visiting history between ages 13 and 30 years and oral health‐related impact. *Community dentistry and oral epidemiology.* 2014;42(3):254-262.

34. Broughton J, Maipi J, Person M, Randall A, Thomson M. Self-reported oral health and dental service-use of rangatahi within the rohe of Tainui. *New Zealand Dental Journal.* 2012;108(3):90.

35. Busato IMS, Ignácio SA, Brancher JA, Grégio AMT, Machado MÂN, Azevedo-Alanis LR. Impact of xerostomia on the quality of life of adolescents with type 1 diabetes mellitus. *Oral Surgery, Oral Medicine, Oral Pathology, Oral Radiology, and Endodontology.* 2009;108(3):376-382.

36. Busato IMS, Ignácio SA, Brancher JA, Moysés ST, Azevedo‐Alanis LR. Impact of clinical status and salivary conditions on xerostomia and oral health‐related quality of life of adolescents with type 1 diabetes mellitus. *Community dentistry and oral epidemiology.* 2012;40(1):62-69.

37. Busato IMS, Thomaz M, Toda AI, et al. Prevalence and impact of xerostomia on the quality of life of people living with HIV/AIDS from Brazil. *Special Care in Dentistry.* 2013;33(3):128-132.

38. Caglayan F, Altun O, Miloglu O, Kaya M-D, Yilmaz A-B. Correlation between oral health-related quality of life (OHQoL) and oral disorders in a Turkish patient population. *Med Oral Patol Oral Cir Bucal.* 2009;14(11):e573-e578.

39. Carvalho JC, Mestrinho HD, Stevens S, Van Wijk AJ. Do oral health conditions adversely impact young adults? *Caries research.* 2015;49(3):266-274.

40. Cassetta M, Altieri F. The influence of mandibular third molar germectomy on the treatment time of impacted mandibular second molars using brass wire: a prospective clinical pilot study. *International journal of oral and maxillofacial surgery.* 2017;46(7):905-911.

41. Cassetta M, Di Carlo S, Giansanti M, Pompa V, Pompa G, Barbato E. The impact of osteotomy technique for corticotomy-assisted orthodontic treatment (CAOT) on oral health-related quality of life. *Eur Rev Med Pharmacol Sci.* 2012;16(12):35-40.

42. Chen M, Wang D-W, Wu L-P. Fixed orthodontic appliance therapy and its impact on oral health-related quality of life in Chinese patients. *The Angle Orthodontist.* 2010;80(1):49-53.

43. Colussi PRG, Hugo FN, Muniz FWMG, Rösing CK. Oral health-related quality of life and associated factors in Brazilian adolescents. *Brazilian dental journal.* 2017;28(1):113-120.

44. Crocombe LA, Mahoney GD. Does optimal access to dental care counteract the oral health‐related quality of life social gradient? *Australian dental journal.* 2016;61(4):418-424.

45. De Oliveira CM, Sheiham A. The relationship between normative orthodontic treatment need and oral health‐related quality of life. *Community dentistry and oral epidemiology.* 2003;31(6):426-436.

46. De Oliveira C, Sheiham A. Orthodontic treatment and its impact on oral health-related quality of life in Brazilian adolescents. *Journal of orthodontics.* 2004;31(1):20-27.

47. Fernandes MJ, Ruta DA, Ogden GR, Pitts NB, Ogston SA. Assessing oral health‐related quality of life in general dental practice in Scotland: validation of the OHIP‐14. *Community dentistry and oral epidemiology.* 2006;34(1):53-62.

48. Feu D, de Oliveira BH, de Oliveira Almeida MA, Kiyak HA, Miguel JA. Oral health-related quality of life and orthodontic treatment seeking. *American journal of orthodontics and dentofacial orthopedics : official publication of the American Association of Orthodontists, its constituent societies, and the American Board of Orthodontics.* 2010;138(2):152-159.

49. Feu D, Miguel JA, Celeste RK, Oliveira BH. Effect of orthodontic treatment on oral health-related quality of life. *Angle Orthod.* 2013;83(5):892-898.

50. Giannetti L, Murri A, Vecci F, Gatto R. Dental avulsion: therapeutic protocols and oral health-related quality of life. *European journal of paediatric dentistry.* 2007;8(2):69-75.

51. Goelzer JG, Becker OE, Haas Junior OL, et al. Assessing change in quality of life using the Oral Health Impact Profile (OHIP) in patients with different dentofacial deformities undergoing orthognathic surgery: a before and after comparison. *Int J Oral Maxillofac Surg.* 2014;43(11):1352-1359.

52. Goh V, Nihalani D, Yeung KWS, Corbet EF, Leung WK. Moderate- to long-term therapeutic outcomes of treated aggressive periodontitis patients without regular supportive care. *Journal of periodontal research.* 2018;53(3):324-333.

53. Hanisch M, Wiemann S, Jung S, Kleinheinz J, Bohner L. Oral Health-Related Quality of Life in People with Rare Hereditary Connective Tissue Disorders: Marfan Syndrome. *International journal of environmental research and public health.* 2018;15(11).

54. He SL, Wang JH. Validation of the chinese version of the oral health impact profile for TMDs (OHIP- TMDs-C). *Med Oral Patol Oral Cir Bucal.* 2015;20(2):e161-166.

55. Hongxing L, List T, Nilsson M, Johansson A, Astrøm AN. Validity and reliability of OIDP and OHIP-14: a survey of Chinese high school students. *BMC oral health.* 2014;14(1):158.

56. Ishida S, Shibuya Y, Kobayashi M, Komori T. Assessing stomatognathic performance after mandibulectomy according to the method of mandibular reconstruction. *International journal of oral and maxillofacial surgery.* 2015;44(8):948-955.

57. Jamilian A, Kiaee B, Sanayei S, Khosravi S, Perillo L. Orthodontic treatment of malocclusion and its impact on oral health-related quality of life. *The open dentistry journal.* 2016;10:236.

58. John MT, Hujoel P, Miglioretti DL, LeResche L, Koepsell TD, Micheelis W. Dimensions of Oral-health-related Quality of Life. *Journal of dental research.* 2004;83(12):956-960.

59. Keles S, Abacigil F, Adana F. Oral health status and oral health related quality of life in adolescent workers. *Clujul Medical.* 2018;91(4):462.

60. Kenig N, Nikolovska J. Assessing the psychometric characteristics of the Macedonian version of the Oral Health Impact Profile questionnaire (OHIP-MAC49). *Oral Health Dent Manag.* 2012;11(1):29-38.

61. Khalifa N, Allen PF, Abu-bakr NH, Abdel-Rahman ME. Psychometric properties and performance of the Oral Health Impact Profile (OHIP-14s-ar) among Sudanese adults. *Journal of oral science.* 2013;55(2):123-132.

62. Kilinc A, Ertas U. An assessment of the quality of life of patients with class III deformities treated with orthognathic surgery. *Journal of Oral and Maxillofacial Surgery.* 2015;73(7):1394. e1391-1394. e1395.

63. Kürklü-Gürleyen E, Öğüt-Erişen M, Çakır O, Uysal Ö, Ak G. Quality of life in patients with recurrent aphthous stomatitis treated with a mucoadhesive patch containing citrus essential oil. *Patient preference and adherence.* 2016;10:967.

64. Lee S, McGrath C, Samman N. Quality of life in patients with dentofacial deformity: a comparison of measurement approaches. *International journal of oral and maxillofacial surgery.* 2007;36(6):488-492.

65. Levin L, Zini A, Levine J, et al. Dental anxiety and oral health-related quality of life in aggressive periodontitis patients. *Clinical oral investigations.* 2018;22(3):1411-1422.

66. Lewandowski B, Szeliga E, Czenczek-Lewandowska E, et al. Comparison of oral-health-related quality of life in patients in the short-and long-term period following lower-facial injury and fractures–preliminary report. *Dental and medical problems.* 2018;55(1):57-62.

67. Li X, Zhu K, Liu F, Li H. Assessment of quality of life in giant ameloblastoma adolescent patients who have had mandible defects reconstructed with a free fibula flap. *World journal of surgical oncology.* 2014;12(1):201.

68. Liu L-J, Xiao W, He Q-B, Jiang W-W. Generic and oral quality of life is affected by oral mucosal diseases. *BMC Oral Health.* 2012;12(1):2.

69. Liu Z, McGrath C, Hägg U. Associations between orthodontic treatment need and oral health‐related quality of life among young adults: does it depend on how you assess them? *Community dentistry and oral epidemiology.* 2011;39(2):137-144.

70. Lopez R, Baelum V. Spanish version of the oral health impact profile (OHIP-Sp). *BMC Oral Health.* 2006;6(1):11.

71. López R, Baelum V. Oral health impact of periodontal diseases in adolescents. *Journal of dental research.* 2007;86(11):1105-1109.

72. Maia CdVR, Mendes FM, Normando D. The impact of oral health on quality of life of urban and riverine populations of the Amazon: A multilevel analysis. *PloS one.* 2018;13(11).

73. Manjith C, Karnam SK, Manglam S, Praveen M, Mathur A. Oral Health-Related Quality of Life (OHQoL) among adolescents seeking orthodontic treatment. *J Contemp Dent Pract.* 2012;13(3):294-298.

74. Mary AV, Mahendra J, John J, Moses J, Ebenezar AR, Kesavan R. Assessing quality of life using the oral health impact profile (OHIP-14) in subjects with and without orthodontic treatment need in chennai, tamil nadu, India. *Journal of clinical and diagnostic research: JCDR.* 2017;11(8):ZC78.

75. Masood M, Masood Y, Newton T. Cross-bite and oral health related quality of life in young people. *Journal of dentistry.* 2014;42(3):249-255.

76. Masood M, Masood Y, Newton T, Lahti S. Development of a conceptual model of oral health for malocclusion patients. *The Angle Orthodontist.* 2015;85(6):1057-1063.

77. Obilade OA, Sanu OO, da Costa OO. Impact of three malocclusion traits on the quality of life of orthodontic patients. *International orthodontics.* 2016;14(3):366-385.

78. Oliveira DC, Ferreira FM, Morosini IdA, Torres-Pereira CC, Martins Paiva S, Fraiz FC. Impact of oral health status on the oral health-related quality of life of Brazilian male incarcerated adolescents. *Oral Health Prev Dent.* 2015;13(5):417-425.

79. Lundgren GP, Karsten A, Dahllöf G. Oral health-related quality of life before and after crown therapy in young patients with amelogenesis imperfecta. *Health and quality of life outcomes.* 2015;13(1):197.

80. Ravaghi V, Mokhatry Ardakan M, Shahriari S, Mokhtari N, Underwood M. Comparison of the COHIP and OHIP-14 as measures of the oral health-related quality of life of adolescents. *Community dental health.* 2011;28(1):82.

81. Rimal J, Shrestha A. Validation of Nepalese Oral Health Impact Profile14 and Assessment of Its Impact in Patients with Oral Submucous Fibrosis in Nepal. *Journal of Nepal Health Research Council.* 2015;13(29):43-49.

82. Sampogna F, Söderfeldt B, Axtelius B, et al. Comparison of patients' and providers' severity evaluation of oral mucosal conditions. *Journal of the American Academy of Dermatology.* 2011;65(1):69-76.

83. Santa-Rosa TTDA, Ferreira RC, Drummond AMA, De Magalhães CS, Vargas AMD, Ferreira EFE. Impact of aesthetic restorative treatment on anterior teeth with fluorosis among residents of an endemic area in Brazil: intervention study. *BMC oral health.* 2014;14(1):52.

84. Saub R, Locker D, Allison P. Comparison of two methods in deriving a short version of oral health-related quality of life measure. *Community dental health.* 2008;25(3):132-136.

85. Schmidt A, Ciesielski R, Orthuber W, Koos B. Survey of oral health-related quality of life among skeletal malocclusion patients following orthodontic treatment and orthognathic surgery. *Journal of Orofacial Orthopedics/Fortschritte der Kieferorthopädie.* 2013;74(4):287-294.

86. Siluvai S, Kshetrimayum N, Reddy C, Siddanna S, Manjunath M, Rudraswamy S. Malocclusion and related quality of life among 13-to 19-year-old students in Mysore City-a cross-sectional study. *Oral Health Prev Dent.* 2015;13(2):135-141.

87. Silva I, Cardemil C, Kashani H, et al. Quality of life in patients undergoing orthognathic surgery–A two-centered Swedish study. *Journal of Cranio-Maxillofacial Surgery.* 2016;44(8):973-978.

88. Silva L, Thomaz E, Freitas HV, Pereira A, Ribeiro C, Alves C. Impact of Malocclusion on the Quality of Life of Brazilian Adolescents: A Population-Based Study. *Plos one.* 2016;11(9):e0162715-e0162715.

89. Silveira MF, Marôco JP, Freire RS, Martins AMEdB, Marcopito LF. Impact of oral health on physical and psychosocial dimensions: an analysis using structural equation modeling. *Cadernos de saude publica.* 2014;30:1169-1182.

90. Soe K, Gelbier S, Robinson P. Reliability and validity of two oral health related quality of life measures in Myanmar adolescents. *Community dental health.* 2004;21(4):306-311.

91. Stange KM, Lindsten R, Bjerklin K. Autotransplantation of premolars to the maxillary incisor region: a long-term follow-up of 12–22 years. *European journal of orthodontics.* 2016;38(5):508-515.

92. Stelzle F, Rohde M, Oetter N, et al. Gingival esthetics and oral health-related quality of life in patients with cleft lip and palate. *International journal of oral and maxillofacial surgery.* 2017;46(8):993-999.

93. Terheyden H, Wüsthoff F. Occlusal rehabilitation in patients with congenitally missing teeth—dental implants, conventional prosthetics, tooth autotransplants, and preservation of deciduous teeth—a systematic review. *International journal of implant dentistry.* 2015;1(1):30.

94. Tinoco-Araujo J, Orti-Raduan E, Santos D, et al. Oral health-related quality of life before hematopoietic stem cell transplantation. *Clinical oral investigations.* 2015;19(9):2345-2349.

95. Van Lierde K, Luyten A, D'haeseleer E, et al. Articulation and oromyofunctional behavior in children seeking orthodontic treatment. *Oral diseases.* 2015;21(4):483-492.

96. Yu D, Wang F, Wang X, Fang B, Shen SG. Presurgical motivations, self-esteem, and oral health of orthognathic surgery patients. *Journal of Craniofacial Surgery.* 2013;24(3):743-747.

97. Yule PL, Durham J, Playford H, et al. OHIP‐TMD s: a patient‐reported outcome measure for temporomandibular disorders. *Community dentistry and oral epidemiology.* 2015;43(5):461-470.

98. Zanatta FB, Ardenghi TM, Antoniazzi RP, Pinto TMP, Rösing CK. Association between gingival bleeding and gingival enlargement and oral health-related quality of life (OHRQoL) of subjects under fixed orthodontic treatment: a cross-sectional study. *BMC Oral Health.* 2012;12(1):53.

99. Zheng D-H, Wang X-X, Su Y-R, et al. Assessing changes in quality of life using the Oral Health Impact Profile (OHIP) in patients with different classifications of malocclusion during comprehensive orthodontic treatment. *BMC oral health.* 2015;15(1):148.

100. Antoun JS, Thomson WM, Merriman TR, Rongo R, Farella M. Impact of skeletal divergence on oral health-related quality of life and self-reported jaw function. *The Korean Journal of Orthodontics.* 2017;47(3):186-194.

101. Antoun JS, Fowler PV, Jack HC, Farella M. Oral health–related quality of life changes in standard, cleft, and surgery patients after orthodontic treatment. *American Journal of Orthodontics and Dentofacial Orthopedics.* 2015;148(4):568-575.

102. Anweigi L, Allen P, Ziada H. The use of the Oral Health Impact Profile to measure the impact of mild, moderate and severe hypodontia on oral health‐related quality of life in young adults. *Journal of oral rehabilitation.* 2013;40(8):603-608.

103. Broder HL, Slade G, Caine R, Reisine S. Perceived impact of oral health conditions among minority adolescents. *Journal of public health dentistry.* 2000;60(3):189-192.

104. Choi S-H, Kim J-S, Cha J-Y, Hwang C-J. Effect of malocclusion severity on oral health–related quality of life and food intake ability in a Korean population. *American Journal of Orthodontics and Dentofacial Orthopedics.* 2016;149(3):384-390.

105. de Paula J, Delcides F, Santos NC, da Silva ÉT, Nunes MF, Leles CR. Psychosocial Impact of Dental Esthetics on Quality of Life in Adolescents: Association with Malocclusion, Self-Image, and Oral Health–Related Issues. *The Angle Orthodontist.* 2009;79(6):1188-1193.

106. Montero J, Costa J, Bica I, Barrios R. Caries and quality of life in portuguese adolescents: Impact of diet and behavioural risk factors. *Journal of clinical and experimental dentistry.* 2018;10(3):e218.

107. Nichols GA, Antoun JS, Fowler PV, Al-Ani AH, Farella M. Long-term changes in oral health-related quality of life of standard, cleft, and surgery patients after orthodontic treatment: A longitudinal study. *American Journal of Orthodontics and Dentofacial Orthopedics.* 2018;153(2):224-231.

108. Oziegbe EO, Esan TA, Adesina BA. Impact of oral conditions on the quality of life of secondary schoolchildren in Nigeria. *Journal of Dentistry for Children.* 2012;79(3):159-164.

109. Papaioannou W, Oulis C, Latsou D, Yfantopoulos J. Oral health related quality of life of Greek adolescents: a cross-sectional study. *European Archives of Paediatric Dentistry.* 2011;12(3):146-150.

110. Roumani T, Oulis CJ, Papagiannopoulou V, Yfantopoulos J. Validation of a Greek version of the oral health impact profile (OHIP-14) in adolescents. *European Archives of Paediatric Dentistry.* 2010;11(5):247-252.

111. Zhou Y, Zheng M, Lin J, Wang Y, Ni ZY. Self-ligating brackets and their impact on oral health-related quality of life in Chinese adolescence patients: a longitudinal prospective study. *The Scientific World Journal.* 2014;2014.
